# Melatonin Regulates Iron Homeostasis by Inducing Hepcidin Expression in Hepatocytes

**DOI:** 10.3390/ijms23073593

**Published:** 2022-03-25

**Authors:** Woo-Ram Park, Byungyoon Choi, Yu-Ji Kim, Yong-Hoon Kim, Min-Jung Park, Dong-Il Kim, Hueng-Sik Choi, Don-Kyu Kim

**Affiliations:** 1 Department of Integrative Food, Bioscience and Biotechnology, Chonnam National University, Gwangju 61186, Korea; 197726@jnu.ac.kr (W.-R.P.); 198347@jun.ac.kr (B.C.); 178252@jnu.ac.kr (Y.-J.K.); 2 Laboratory Animal Resource Center, Korea Research Institute of Bioscience and Biotechnology, Daejeon 34141, Korea; yhoonkim@kribb.re.kr; 3 Department of Physiology, College of Veterinary Medicine, Chonnam National University, Gwangju 61186, Korea; mjpark@jnu.ac.kr (M.-J.P.); kimdi@jnu.ac.kr (D.-I.K.); 4 School of Biological Sciences and Technology, Chonnam National University, Gwangju 61186, Korea; hsc@chonnam.ac.kr

**Keywords:** circadian rhythm, cell signaling, gene regulation, hepcidin

## Abstract

The pineal hormone, melatonin, plays important roles in circadian rhythms and energy metabolism. The hepatic peptide hormone, hepcidin, regulates iron homeostasis by triggering the degradation of ferroportin (FPN), the protein that transfers cellular iron to the blood. However, the role of melatonin in the transcriptional regulation of hepcidin is largely unknown. Here, we showed that melatonin upregulates *hepcidin* gene expression by enhancing the melatonin receptor 1 (MT1)-mediated c-Jun N-terminal kinase (JNK) activation in hepatocytes. Interestingly, *hepcidin* gene expression was increased during the dark cycle in the liver of mice, whereas serum iron levels decreased following hepcidin expression. In addition, melatonin significantly induced *hepcidin* gene expression and secretion, as well as the subsequent FPN degradation in hepatocytes, which resulted in cellular iron accumulation. Melatonin-induced hepcidin expression was significantly decreased by the melatonin receptor antagonist, luzindole, and by the knockdown of MT1. Moreover, melatonin activated JNK signaling and upregulated hepcidin expression, both of which were significantly decreased by SP600125, a specific JNK inhibitor. Chromatin immunoprecipitation analysis showed that luzindole significantly blocked melatonin-induced c-Jun binding to the *hepcidin* promoter. Finally, melatonin induced hepcidin expression and secretion by activating the JNK-c-Jun pathway in mice, which were reversed by the luzindole treatment. These findings reveal a previously unrecognized role of melatonin in the circadian regulation of hepcidin expression and iron homeostasis.

## 1. Introduction

Iron is an essential element not only for vertebrates, but also for most microorganisms, plants, and invertebrates [1]. In mammals, dietary Fe^3+^ iron is reduced to Fe^2+^ by cellular reductase, which is transported to enterocytes via divalent metal transporter-1 [2]. Absorbed iron binds to the iron-transporter protein, transferrin, which circulates through the bloodstream and is imported into the cells by binding to transferrin receptors (Tfr) [3]. Most of the iron in the body is bound to the hemoglobin in red blood cells, and this iron is recycled by macrophages through the degradation of heme by the hydrolytic enzyme heme oxygenase 1 [4].

Hepcidin is an antimicrobial peptide hormone that plays an important role in iron homeostasis [5,6]. It is synthesized and secreted by the liver and binds to the iron exporter ferroportin (FPN) which is present on the membrane of enterocytes, macrophages, and hepatocytes. By binding to FPN, hepcidin inhibits cellular iron export by inducing its internalization and degradation [7]. Transcription of the *hepcidin* gene is regulated by two main stimuli, inflammation and plasma iron concentration [8,9]. Inflammatory signals, such as interleukin-6 (IL-6), activate the Janus kinase/signal transducer and activator of the transcription 3 signaling pathway, resulting in hepcidin induction [10]. IL-6-induced hepcidin inhibits the absorption of dietary iron, resulting in hypoferremia, which regulates pathogen survival [11]. The response of hepcidin to cellular iron levels is regulated by bone morphogenetic protein 6 (BMP6)/SMAD signaling [12]. BMP6 binds to BMP type I and type II serine/threonine kinase receptors and induces phosphorylation of SMAD proteins (SMAD1, SMAD5, and SMAD8) [13]. These findings suggest a critical role of hepcidin in iron metabolism. However, the circadian regulation of hepcidin and iron homeostasis remains unknown.

Melatonin, which is also known as N-acetyl-5-methoxytryptamine, is a tryptophan derivative that is mainly produced by the pineal gland and is dependent on the light/dark cycle [14]. It functions as a strong antioxidant and directly scavenges free radicals, including superoxide, hydrogen peroxide, and nitric oxide [15]. Melatonin also reduces oxidative stress by regulating the transcription and activity of antioxidant enzymes, such as superoxide dismutase, catalase, and glutathione peroxidase [16]. Melatonin binds to melatonin receptor type (MT) 1 and MT2—which are G-protein-coupled receptors—and controls diverse cellular functions such as energy metabolism, cardiac function, and circadian rhythm [17,18,19,20]. It also regulates lipid metabolism by upregulating the expression of genes related to lipolysis [21]. In addition, melatonin increases hypothalamic Akt expression, resulting in the suppression of hepatic gluconeogenesis [22]. These results were further supported by a previous study that reported decreased free fatty acid levels and systemic insulin resistance in MT1 knockout (KO) mice [23]. Melatonin also plays an important role in innate immunity by activating immune cells, such as monocytes, neutrophils, natural killer cells, and macrophages [24]. In addition, melatonin had a protective effect against sepsis caused by bacterial infection [25]. Interestingly, it was reported that high serum iron levels increased mortality in sepsis patients [26]. These findings suggest that there may be a positive correlation between melatonin and iron metabolism. Indeed, reports have shown that melatonin plays a role in iron metabolism. For example, melatonin regulates the concentration of iron-related proteins, including ferritin and transferrin [27]. Moreover, the administration of melatonin decreased the expression of Tfr which mediates the cellular uptake of iron under hypoxia [28]. Recently, it was reported that melatonin inhibits iron-overload-induced apoptosis and necrosis in bone marrow mesenchymal stem cells [29]. However, the mechanistic link between melatonin and iron metabolism is largely unknown. 

In this study, we demonstrated the circadian regulation of hepcidin expression and iron homeostasis. In addition, the pineal hormone, melatonin, regulated the hepcidin expression and iron metabolism by inducing the activation of MT1-mediated c-Jun N-terminal kinase (JNK) signaling in hepatocytes. These findings reveal a previously unrecognized role of melatonin in the transcriptional control of hepcidin—an inducible hepatokine and a key regulator of iron homeostasis.

## 2. Results

### 2.1. Circadian Oscillation of Hepatic Hepcidin Gene Expression

**Figure 1:**
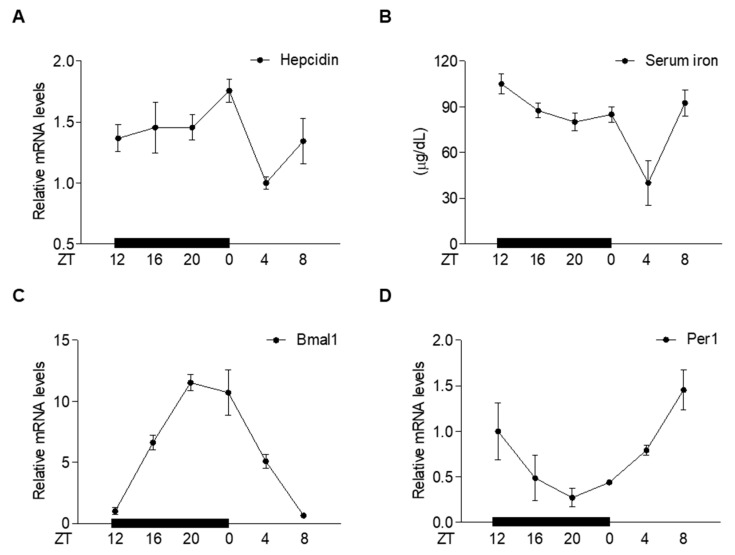
Circadian oscillation of hepatic *hepcidin* gene expression and serum iron levels. (**A**–**D**) C57BL/6J mice (*n* = 5 per ZT point) were sacrificed at different ZT points (ZT0, ZT4, ZT8, ZT12, ZT16, and ZT20) to assess the following: (**A**) *Hepcidin* mRNA levels in the liver; (**B**) serum iron levels; (**C**) *Bmal1* mRNA levels in the liver; (**D**) *Per1* mRNA levels in the liver. All experiments were performed in triplicate and repeated at least three times. Data are presented as means ± SD.

To investigate whether *hepcidin* gene expression in the liver undergoes circadian oscillation, we examined the expression of hepcidin in the liver of mice during a 24 h circadian cycle. Interestingly, *hepcidin* gene expression was increased during the dark cycle and peaked at ZT 0, whereas serum iron levels decreased following hepcidin expression during the dark cycle (Figure 1A,B). Circadian rhythms are controlled by the core circadian clock genes, Bmal1, Clock, Cry, and Per [30]. These genes constitute a transcriptional feedback loop and generate circadian oscillation with a 24 h cycle in which the Clock and Bmal1 heterodimer complex activates the transcription of Cry and Per, which functions as a transcriptional repressor [31]. Similarly, Bmal1 and Per1 exhibited circadian expression patterns and showed negative feedback loops (Figure 1C,D). However, these clock genes did not affect *hepcidin* gene expression (Figure S1). These findings suggest that melatonin could be involved in the circadian oscillation of hepatic hepcidin and serum iron levels.

### 2.2. Melatonin Induces Hepcidin Expression and Secretion

**Figure 2:**
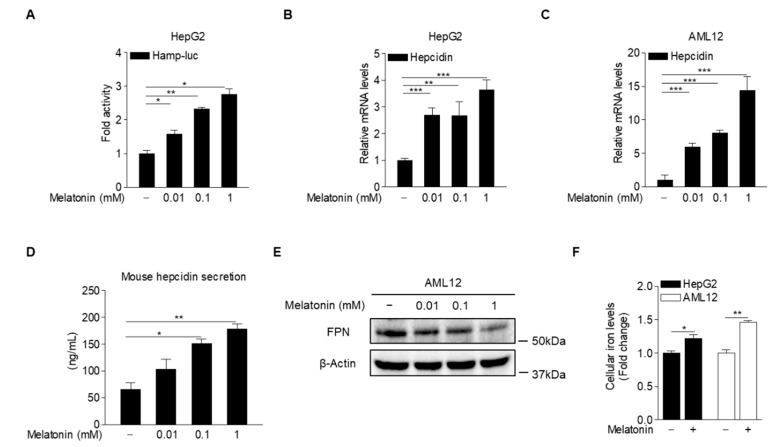
Melatonin upregulates hepcidin production in hepatocytes. (**A**) Effect of melatonin on *hepcidin*-promoter activity. HepG2 cells were transfected with a *hepcidin*-promoter reporter (mHamp-luc; 200 ng) and then treated with melatonin for 12 h after 2 h of serum starvation. (**B**,**C**) Quantitative polymerase chain reaction (qPCR) analysis showing hepcidin mRNA expression in (**B**) HepG2 cells and (**C**) AML12 cells. The cells were treated with melatonin for 6 h after 2 h of serum starvation. (**D**) Melatonin-induced hepcidin secretion. (**E**) Western blot analysis of FPN expression. AML12 cells were treated with melatonin for 6 h after 2 h of serum starvation. (**F**) Cellular iron concentration in HepG2 and AML12 cells treated with melatonin (100 µM) for 6 h after 2 h of serum starvation. Gels for Western blot analysis were run under the same experimental conditions. All experiments were performed in triplicate and repeated at least three times. Data are presented as means ± SD. * *p* < 0.05, ** *p* < 0.01, *** *p* < 0.001 using two-tailed Student’s *t*-test.

To examine the possibility that melatonin may regulate *hepcidin* gene expression in hepatocytes, we analyzed *hepcidin*-promoter activity in HepG2 cells treated with various concentrations of melatonin. As expected, melatonin significantly increased *hepcidin*-promoter activity (Figure 2A). In addition, hepcidin mRNA levels were significantly induced in HepG2 (human hepatoblastoma cell line) and AML12 cells (alpha mouse liver 12, a non-transformed mouse liver cell line) treated with melatonin (Figure 2B,C). To examine the effect of melatonin on hepcidin secretion, we measured the levels of secreted hepcidin in the culture medium of melatonin-treated AML12 cells. The results showed that melatonin significantly increased hepcidin secretion, which was followed by FPN degradation, resulting in a high iron concentration in HepG2 and AML12 cells (Figure 2D–F). These results indicated that melatonin induces hepcidin transcription and secretion in hepatocytes. 

### 2.3. Melatonin Receptor Blockade Prevents Induction of Hepcidin by Melatonin 

**Figure 3:**
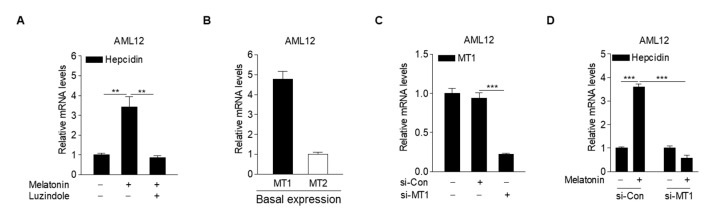
Melatonin increases hepcidin expression through activation of MT1. (**A**) Effect of luzindole on melatonin-induced hepcidin mRNA expression in AML12 cells. The cells were treated with melatonin (100 µM) and luzindole (10 µM) for 6 h after 2 h of serum starvation. (**B**) qPCR analysis showing the basal expression levels of MT1 and MT2 in AML12 cells. (**C**) qPCR analysis showing the knockdown efficiency of MT1 using si-MT1. AML12 cells were transfected with either si-Con or si-MT1 for 48 h. (**D**) The effect of MT1 knockdown on melatonin-induced hepcidin expression. AML12 cells were transfected with si-MT1 and then treated with melatonin (100 µM) for 6 h after 2 h of serum starvation. All experiments were performed in triplicate and repeated at least three times. Data are presented as means ± SD. ** *p* < 0.01, *** *p* < 0.001 using two-tailed Student’s *t*-test.

To elucidate the molecular mechanism underlying melatonin-dependent hepcidin expression, we investigated whether luzindole, a MT antagonist, inhibits melatonin-induced hepcidin expression in hepatocytes. The results showed that luzindole treatment almost completely inhibited the melatonin-induced increase in hepcidin mRNA levels (Figure 3A), implying that melatonin upregulates hepcidin expression by activating MT signaling. Interestingly, we found that basal MT1 expression levels were higher than MT2 expression levels in hepatocytes (Figure 3B). To demonstrate the role of MT1 in the melatonin-mediated increase in hepcidin expression in hepatocytes, AML12 cells transfected with a small-interfering RNA (siRNA) for MT1 (si-MT1) were treated with melatonin. As expected, melatonin-induced hepcidin expression was significantly blocked by transfection with si-MT1 (Figure 3C,D). These results demonstrated that melatonin regulates hepcidin expression by activating MT1 signaling in hepatocytes.

### 2.4. Melatonin Upregulates Hepcidin Expression by Activating the MT1-JNK-c-Jun Pathway

**Figure 4:**
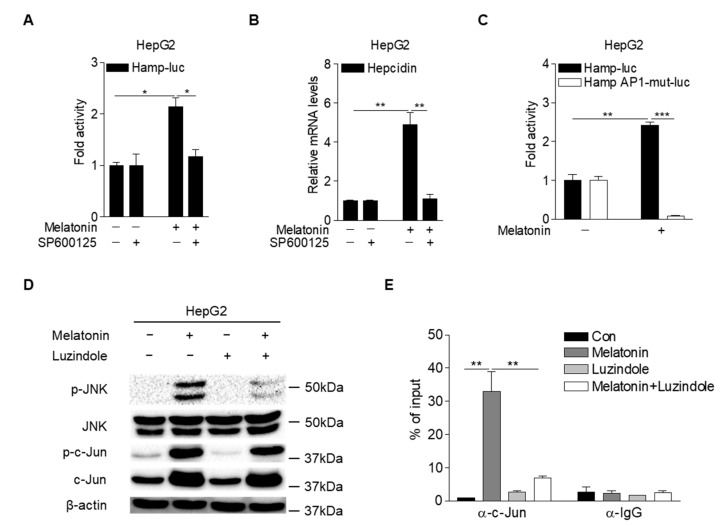
Melatonin activates MT1-JNK signaling in hepatocytes. (**A**) Inhibitory effect of SP600125 (a specific JNK inhibitor) on melatonin-induced *hepcidin*-promoter activity. HepG2 cells were transfected with mHamp-luc (200 ng) and then treated with melatonin (100 µM) and SP600125 (50 µM) for 12 h after 2 h of serum starvation. (**B**) qPCR analysis of hepcidin mRNA expression. HepG2 cells were treated with melatonin (100 µM) and SP600125 (50 µM) for 6 h after 2 h of serum starvation. (**C**) AP-1-dependent regulation of *hepcidin*-promoter activity by melatonin. HepG2 cells were transfected with mHamp-luc (wild-type, 200 ng) or mHamp AP1-mut-luc (200 ng) and then treated with melatonin (100 µM) for 12 h after 2 h of serum starvation. (**D**) Inhibitory effect of luzindole on melatonin-mediated activation of JNK signaling. HepG2 cells were treated with melatonin (100 µM) and luzindole (10 µM) for 6 h after 2 h of serum starvation. (**E**) Chromatin immunoprecipitation assay showing the inhibitory effect of luzindole on melatonin-induced c-Jun binding to the *hepcidin* promoter. Soluble chromatin was immunoprecipitated with an anti-IgG or anti-c-Jun antibody. Gels for Western blot analysis were run under the same experimental conditions. All experiments were performed in triplicate and repeated at least three times. Data are presented as means ± SD. * *p* < 0.05, ** *p* < 0.01, *** *p* < 0.001 using two-tailed Student’s *t*-test.

Lipopolysaccharide induced hepcidin expression by activating JNK-c-Jun signaling in hepatocytes [32]. To investigate whether JNK is involved in melatonin-induced hepcidin expression, we analyzed *hepcidin*-promoter activity and mRNA expression in HepG2 cells treated with melatonin and SP600125, a specific JNK inhibitor. The results showed that melatonin-induced *hepcidin*-promoter activity and mRNA expression were significantly reduced upon treatment with SP600125 (Figure 4A,B). It was reported that JNK-mediated phosphorylation of c-Jun increases AP-1 binding to target gene promoters [33]. Melatonin significantly increased *hepcidin*-promoter activity in HepG2 cells, which was almost completely blocked by the mutation of the AP-1-binding site in the *hepcidin* promoter (Figure 4C). Melatonin-mediated phosphorylation of JNK and c-Jun was significantly decreased in HepG2 cells treated with luzindole (Figure 4D). Furthermore, a chromatin immunoprecipitation (ChIP) assay demonstrated that melatonin increased c-Jun binding to the *hepcidin* promoter, which was significantly blocked by luzindole treatment (Figure 4E). These results suggest that melatonin induces hepcidin expression by activating the JNK-c-Jun pathway in hepatocytes.

### 2.5. Melatonin Regulates Hepcidin Expression in Mice

**Figure 5:**
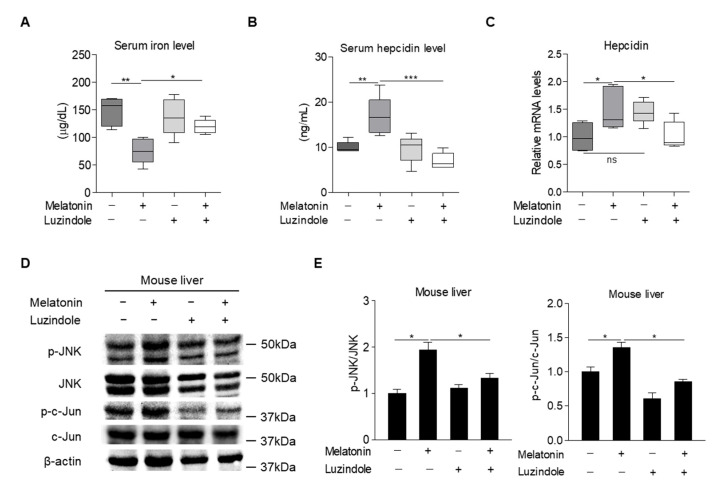
Effects of melatonin on hepcidin expression and iron metabolism in mice. (**A**–**E**) C57BL/6J mice were intraperitoneally injected with melatonin (10 mg/kg, *n* = 5) and luzindole (10 mg/kg, *n* = 5) for 12 h and then the following were analyzed: (**A**) Serum iron levels; (**B**) serum hepcidin levels; (**C**) hepcidin mRNA expression in the liver; and (**D**) Western blot analysis. (**E**) Graphical representation showing JNK and c-Jun protein levels. Gels for Western blot analysis were run under the same experimental conditions. All experiments were performed in triplicate and repeated at least three times. Data are presented as means ± SEM. * *p* < 0.05, ** *p* < 0.01, *** *p* < 0.001 using two-tailed Student’s *t*-test.

Based on the findings in cultured hepatocytes, we next examined whether melatonin could regulate hepcidin expression and secretion, resulting in alteration of iron metabolism in mice. The results showed that melatonin treatment led to a significant increase in hepcidin levels in the liver and serum, concomitant with reduced serum iron levels. Consistent with these findings, melatonin significantly increased the phosphorylation levels of JNK and c-Jun in the liver (Figure 5A–E). Luzindole treatment significantly reversed these effects of melatonin on hepcidin and serum iron levels and on JNK-c-Jun signaling (Figure 5A–E). Thus, we concluded that melatonin upregulates the transcription of hepcidin and thereby alters iron metabolism by activating JNK-c-Jun signaling in hepatocytes.

## 3. Discussion

**Figure 6:**
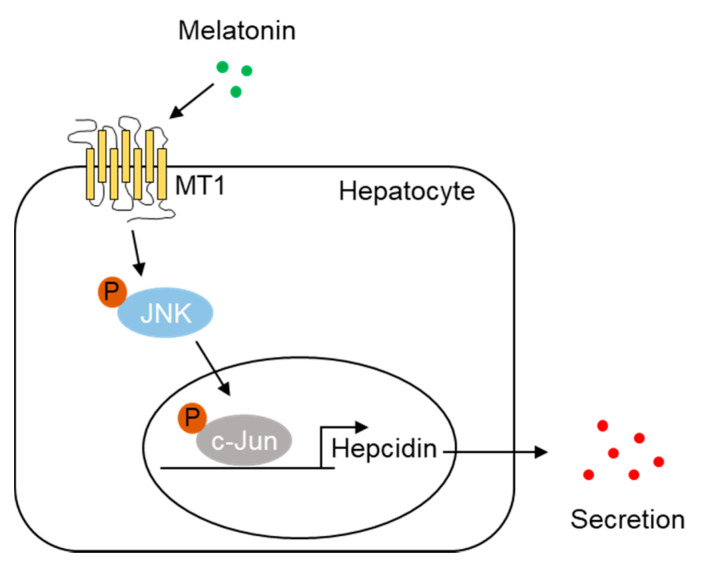
Proposed model for the role of melatonin in hepcidin expression and iron metabolism. Melatonin activates MT1-mediated JNK-c-Jun signaling in hepatocytes and increases hepcidin expression, leading to alteration of iron metabolism.

Hepcidin, which is mostly produced by the liver, controls cellular iron homeostasis by binding to FPN, an iron transporter found in enterocytes, macrophages, and hepatocytes. Under iron overload, intracellular accumulation of iron increases reactive oxygen species (ROS), resulting in cell death via apoptosis, ferroptosis, and necrosis. Recently, it was reported that mitochondrial ROS, produced in response to cellular iron, activates the expression of nuclear factor erythroid 2-related factor (Nrf2) and induces the expression of BMP6 and hepcidin, leading to decreased serum iron levels [34]. These results indicate that hepcidin expression depends on the cellular redox state and is induced by antioxidant effects. These findings are supported by a previous report which showed that resveratrol, a polyphenol, induces hepcidin expression via the Nrf2-c-Jun pathway [35]. In this study, we found that melatonin increased hepcidin expression in a concentration-dependent manner, suggesting that the antioxidant effect of melatonin contributes to hepcidin expression. Indeed, melatonin induced hepcidin transcription and secretion in hepatocytes, whereas a loss of MT1 expression inhibited the melatonin-mediated upregulation of hepcidin. Melatonin also induced the activation of cellular JNK and binding of c-Jun to the AP-1 site of the *hepcidin* promoter, as was demonstrated by the lack of transcriptional upregulation with an AP-1 mutant promoter and a ChIP assay in hepatocytes. Luzindole had inhibitory effects against melatonin-induced hepcidin expression in cultured hepatocytes and mouse livers. Thus, we concluded that melatonin regulates *hepcidin* gene expression through the MT1-JNK-c-Jun signaling pathway (Figure 6).

The circadian clock, which is composed of the suprachiasmatic nucleus and a transcription–translation feedback loop, is influenced by fasting/feeding and the sleep/wake cycle [36]. The circadian clock controls diverse behavioral and physiological processes. For example, glucose, lipid, and bile acid metabolism are regulated by the circadian clock in the liver [37]. The pineal hormone melatonin, which has a circadian rhythm that depends on light/dark signals, also regulates metabolism. It has been reported that melatonin signaling regulates glucose rhythms [38]. Interestingly, we showed here that hepatic hepcidin expression shows a circadian rhythm that is similar to the pattern of mouse melatonin secretion [39]. In addition, we demonstrated that melatonin signaling induced hepcidin expression, leading to hypoferremia in mice. These findings suggest that melatonin acts as a circadian regulator of iron metabolism. However, there are other factors that may affect the circadian rhythm of hepcidin expression. For example, hepcidin expression is regulated by orphan nuclear receptors, such as estrogen related receptor γ (ERRγ) [40] and small heterodimer partner [41], which also showed circadian rhythms in the liver [42]. In addition, insulin and glucose also regulate the concentration of hepcidin [43,44]. Therefore, the detailed molecular mechanism regulating the circadian rhythm of iron metabolism needs to be further characterized.

Under inflammation, the pro-inflammatory cytokine IL-6 induces expression of the orphan nuclear receptor ERRγ and leads to hepcidin secretion [45]. Hepcidin is an antimicrobial peptide hormone that reduces the plasma iron concentration by inducing the degradation of FPN [7] and inhibits the growth and proliferation of extracellular pathogens. It has been reported that overexpression of hepcidin led to a marked reduction in *Plasmodium berghei* infection which causes malaria [46]. Melatonin has also been shown to have a protective effect against sepsis [25]. In this study, we demonstrated that melatonin induced hepcidin production, suggesting that melatonin may regulate bacterial infections by modulating hepcidin expression. However, hepcidin is not always beneficial to the host during pathogen invasion. For example, an increase in intracellular iron promotes the growth of intracellular bacteria, such as *Salmonella enterica*, *Chlamydia psittaci*, and *Legionella pneumophila* [47,48]. Previously, we reported that ERRγ modulates the survival of *Salmonella typhimurium* by regulating hepcidin expression in macrophages [40]. Here, we showed that an intraperitoneal injection of melatonin increased hepcidin production and decreased serum iron levels, which suggests that melatonin may not be effective against intracellular bacterial infection. These findings were further supported by results from a previous study, showing that exogenous melatonin exacerbates *Salmonella enteritidis* infection in molted layers [49]. Interestingly, GSK5182, an inverse agonist of ERRγ, controlled the growth of *S. typhimurium* by decreasing hepcidin expression [40]. In this study, we found that luzindole, a MT antagonist, inhibited melatonin-induced hepcidin expression. Together, these findings suggest that luzindole may have therapeutic effects against intracellular bacterial infection.

## 4. Materials and Methods

### 4.1. Chemicals

Melatonin and luzindole were purchased from Sigma-Aldrich (St. Louis, MO, USA) and then dissolved in ethanol and dimethyl sulfoxide (DMSO), respectively. SP600125 was dissolved in DMSO, as described previously [32]. 

### 4.2. Plasmid DNAs

The mouse *hepcidin*-promoter (mHepcidin-luc, −982/+84 bp) and AP-1 mutant mouse *hepcidin*-promoter (mHepcidin AP1 mut-luc) constructs were described previously [32]. pcDNA3-Flag-mERRγ was described previously [50]. pCMV-SPORT6-Bmal1 and pCMV-SPROTT6-Clock were purchased from Korea Human Gene Bank (Medical Genomics Research Center, KRIBB, Daejeon, Korea).

### 4.3. Animal Experiments

Male, 8-week-old C57BL/6J mice (Jackson Laboratory, Bar Harbor, ME, USA) were used in this study. The mice were acclimatized to a 12 h light/dark cycle, at 22 ± 2 °C, with free access to food and water, in a specific-pathogen-free facility. For the circadian experiments, zeitgeber time (ZT) 0 was lights on, and ZT 12 was lights off for animals under a 12 h light/dark cycle. We also indicated time by the a.m. and p.m. designations, whereby ZT 0 (lights on) was 7:00 a.m., and ZT 12 (lights off) was 7:00 p.m. To investigate the effects of melatonin and luzindole on hepcidin expression, mice were injected with (intraperitoneally (i.p.)) melatonin (10 mg/kg, *n* = 7), luzindole (10 mg/kg, *n* = 7), or melatonin plus luzindole (*n* = 7). Mice were injected with melatonin for 12 h after 4 h of luzindole injection. All experimental procedures were reviewed and approved by the Institutional Animal Care and Use Committee of Chonnam National University (CNU IACUC-YB-R-2021-121). 

### 4.4. Cell Culture and Transient Transfection

HepG2 was cultured in high-glucose Dulbecco’s Modified Eagle’s Medium (DMEM; Welgene, Gyeongsan, Korea) supplemented with 10% heat-inactivated fetal bovine serum (FBS; Gibco, Waltham, MA, USA) and 1% antibiotics (penicillin–streptomycin; Capricorn Scientific, Ebsdorfergrund, Germany). AML12 cells were cultured in DMEM F-12 medium (Welgene) supplemented with 10% FBS, 1% insulin–transferrin–selenium–pyruvate (Welgene), 40 ng/mL dexamethasone, and 1% antibiotics. All cell lines were cultured in a humidified atmosphere containing 5% CO_2_ at 37 °C. Transient transfections were performed using polyethylenimine (Polysciences, Inc., Warrington, PA, USA) or SuperFect reagent (QIAGEN, Hilden, Germany) according to the manufacturer’s instructions. For luciferase assays, the Nano-Glo vector (Promega, Madison, WI, USA) was used as an internal control, and firefly luciferase activity was normalized to Nano-Glo luciferase activity. The data were from at least three independent experiments.

### 4.5. RNA Interference

si-MT1 and si-Con were purchased from QIAGEN (Cat # 1027416). AML12 cells were transfected with si-Con and si-MT1 using Lipofectamine RNAi MAX (Thermo Fisher Scientific, Waltham, MA, USA) according to the manufacturer’s instructions.

### 4.6. Q-PCR Analysis

Total RNA was extracted from cultured hepatocytes or mouse liver tissue using Tri-RNA reagent (Favorgen Biotech Corporation, Ping-Tung, Taiwan) according to the manufacturer’s instructions. The quantity and purity of the extracted RNA were measured using a Biophotometer D30 (Eppendorf, Hamburg, Germany). cDNAs generated using TOPscript RT DryMIX (Enzynomics, Daejeon, Korea) were analyzed with a CFX Connect real-time system (Bio-Rad, Hercules, CA, USA) using TOPreal qPCR 2× PreMIX (SYBR Green with low ROX) (Enzynomics). The results were normalized to the expression of the ribosomal protein L32, and relative gene expression data were analyzed using the delta delta Ct method. All primers used for qPCR analysis are listed in Supplementary Table S1.

### 4.7. Western Blot Analysis

Western blotting was performed with whole-cell extracts and mouse liver tissue and was generated using RIPA buffer (Thermo Fisher Scientific) as previously described [50]. Proteins were separated by 10% SDS-PAGE and then transferred to nitrocellulose membranes (GE Healthcare, Chicago, IL, USA). The primary antibodies used for the immunoblotting assays were anti-JNK (1:2000, Cell Signaling Technology, Danvers, MA, USA), anti-phospho-JNK (1:2000, Cell Signaling Technology), anti-c-Jun (1:2000, Cell Signaling Technology), anti-phospho-c-Jun (1:2000, Cell Signaling Technology), anti-FPN (1:2000, Novus Biologicals, Centennial, CO, USA), and anti-β-Actin (1:3000, Santa Cruz Biotechnology, Dallas, TX, USA). The primary antibodies were probed with HRP-conjugated secondary antibodies (Bethyl Laboratories, Montgomery, TX, USA) and visualized using Amersham ECL Western Blotting Detection Reagent (GE Healthcare). Images were visualized using a Chemi-doc XRS system (Bio-Rad Chemidoc XRS Gel Imaging System).

### 4.8. Measurement of Hepcidin and Serum Iron Levels

Blood samples were collected from mice under anesthesia before killing via intracardiac puncture. Serum hepcidin was measured using the mouse hepcidin enzyme-linked immunosorbent assay (ELISA) kit (Elabscience, Houston, TX, USA) according to the manufacturer’s protocols. AML12 cells were treated with melatonin for 6 h after 2 h of serum starvation. Hepcidin concentrations in the cell culture medium were measured using the mouse hepcidin ELISA kit (Elabscience) according to the manufacturer’s instructions. Serum and cellular iron were measured using a spectrophotometric method (TBA-200FR NEO) or an iron assay kit (Abcam, Cambridge, UK), according to the manufacturer’s instructions.

### 4.9. ChIP Assay

A ChIP assay was performed using the SimpleChIP Plus Enzymatic Chromatin IP kit (Cell Signaling Technology) according to the manufacturer’s protocol. In brief, HepG2 cells were transfected with a mouse *hepcidin*-promoter luciferase reporter construct (mHamp-luc, 4 μg) and then treated with melatonin (1 mM) and luzindole (10 µM) for 6 h after 2 h of serum starvation. Cells were fixed with 1% formaldehyde and then harvested. Soluble chromatin was immunoprecipitated using anti-IgG and anti-c-Jun antibodies (Cell Signaling Technology). After DNA extraction, qPCR was carried out using primers to amplify the AP-1 binding regions on the mouse *hepcidin* promoter. The primers used for qPCR analysis are listed in Supplementary Table S1.

### 4.10. Statistical Analysis

Statistical analyses were performed using GraphPad Prism [51]. Data are presented as means ± standard deviation (SD) or ± standard error of the means (SEM). Significance was determined using a two-tailed Student’s *t*-test (*p* < 0.05).

## 5. Conclusions

In conclusion, we report a previously unrecognized role of melatonin in the circadian regulation of hepcidin expression and iron metabolism, with detailed molecular mechanisms in hepatocytes. Melatonin induced hepatic hepcidin production by enhancing MT1-mediated JNK-c-Jun activation. Thus, these findings suggest that melatonin plays a pivotal role in the circadian regulation of hepcidin and iron homeostasis as well as controlling pathogen invasion.

## Data Availability

Data are contained within the article and Supplementary Material.

## Supplementary Materials

The following are available online at https://www.mdpi.com/article/10.3390/ijms23073593/s1.
