# Supplementary material for: Melatonin Regulates Iron Homeostasis by Inducing Hepcidin Expression in Hepatocytes
Source: Int J Mol Sci. 2022 Mar 25;23(7):3593. doi: 10.3390/ijms23073593 (PMC8998539; doi:10.3390/ijms23073593)
Supplement: Supplementary file 1 [file ijms-23-03593-s001.zip › ijms-1654640-supplementary.pdf]

Supplementary Figure S1

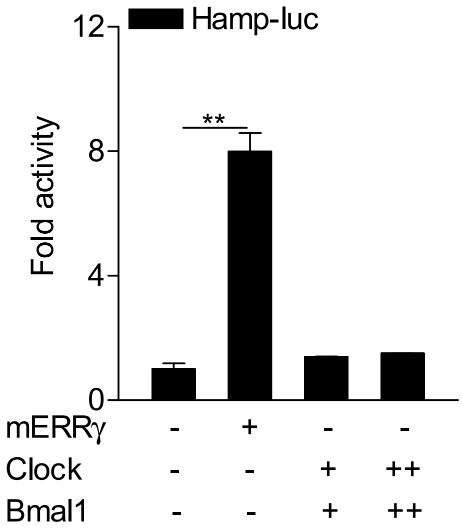

Figure S1. Circadian clock genes did not alter hepcidin promoter activity. HepG2 cells were co-transfected with mHamp-luc (200 ng) and vectors expressing Bmal1 (+: 100 ng, ++: 300 ng), Clock (+: 100 ng, ++: 300 ng) and mERRγ (+: 100 ng, as a positive control). Data are presented as means ± SD. ** $P < 0.01$ using two-tailed Student's *t*-test.

Table S1. Q-PCR primer sequence and accession number for genes used in this study.

Gene name	Forwad (5'-3')	Reverse(5'-3')	Accession number
Bmal1	AACCTTCCCGCAGCTAACAG	AGTCCTCTTTGGGCCACCTT	XM_030242022.2
Per1	GCTTCGTGGACTTGACACCT	TGCTTTAGATCGGCAGTGGT	NM_011065.5
Hepcidin	TGCCTGTCTCCTGCTTCTCCT	GATGGGGAAGTTGGTGTCTC	NM_032541.2
MT1	TACACTGTCAAGTCAGCGCA	GGGCATGATGGCGATGAG	NM_008639.3
MT2	ATCCAGACAGCCAGCACA	TGCGCAAATCACTCGGTC	NM_145712.2
L32	TCTGGTGAAGCCCAAGATCG	CTCTGGGTTTCCGCCAGT	NM_172086.2
Chip-AP-1	CTGGCTGTAGGTGACACAAC	AAGGACTTGTGTGGTGGCTG	
mERRγ			NM_001357534.1
Clock			XM_030254102.2
